# Bibliometric Analysis of Research Relating to Perineal Pain Reported over the Period 1981 to 2021

**DOI:** 10.3390/jpm13030542

**Published:** 2023-03-17

**Authors:** Huang Ding, Qin Chen, Huiming Zhan, Yifan Jia, Juan Ren, Jishi Ye

**Affiliations:** 1 Department of Pain, Renmin Hospital of Wuhan University, Wuhan 430060, China; 2 Operation Room, Hubei Cancer Hospital, Tongji Medical College, Huazhong University of Science and Technology, Wuhan 430060, China; 3 Department of Anesthesiology, The Central Hospital of Wuhan, Tongji Medical College, Huazhong University of Science and Technology, Wuhan 430014, China; 4 Department of Respiratory Medicine, General Hospital of Central Theater Command, Wuhan 430070, China

**Keywords:** bibliometric analysis, perineal pain, research evaluation studies, VOSviewer, Web of Science

## Abstract

Background: Perineal pain is a painful neuropathic condition, which does not have a standard diagnostic or treatment approach. As such, we sought to evaluate the global scientific output of research into perineal pain and explore trends from 1981 to 2021 using bibliometric methods. Methods: Articles on perineal pain were retrieved from the Web of Science (WoS) database. We analyzed the content and quality of publications from within the specified timeframe. We also utilized VOSviewer to mine and cluster data from retrieved articles. Results: A total of 1917 articles were collected. The number of related papers published increased year by year. Articles were most frequently published by authors in the United States and France. Although the US remains at the center of this field, publications from China have become more frequent in recent years. We also found that French academic institutions dominate the field of perineal pain, and Jean-Jacques Labat from Nantes Universite is the most published author in the field. “Episiotomy”, “pain”, “management”, “prostatectomy”, “pelvic pain”, and “complication” were frequently cited as keywords. Conclusion: The increasing number of publications each year indicates that perineal pain has gained more attention as an important research topic.

## 1. Introduction

Perineal pain is a neuropathic condition involving the lower abdomen, including the urinary system, reproductive system, and perianal area, for which there is no diagnostic or treatment standard [1]. Although patients with perineal pain have no specific disease, their symptoms often seriously affect quality of life and may cause psychological and psychiatric disorders. In these cases, the trigger is not the immediate cause of the pain, but rather the painful decompensation of pre-existing nerve compression. Perineal pain can also occur gradually without an established trigger or through a series of spontaneously resolved pain episodes [2]. Unfortunately, there are few clinical and basic studies on the causes of or treatment for perineal pain. Existing pain management programs also lack the support of clinical data from large samples of multi-centers and are controversial. Accordingly, we sought to review the literature for studies of perineal pain research between 1981 and 2021 to identify key research topics and trends.

In recent years, bibliometric analyses have been widely used to assess the credibility, quality, and impact of academic work in multiple research areas, including organoids [3], neurology [4], and oncology [5]. VOSviewer, a bibliometric visualization tool, is widely used to visualize and analyze emerging trends and hot topics in academic papers. These networks may for instance include journals, researchers, or individual publications, and they can be constructed based on citation, bibliographic coupling, co-citation, or co-authorship relationships. VOSviewer also offers text mining functionality that can be used to construct and visualize co-occurrence networks of important terms extracted from a body of scientific literature. [6,7,8]. To date, few bibliometric studies have been performed on research relating to perineal pain. To fill this knowledge gap and gain insights into research trends and hot topics in the field, we performed a bibliometric analysis of research on perineal pain using the Web of Science (WoS) database (https://www.webofscience.com/, accessed on 1 July 2021) and VOSviewer software, version 1.6.18 (Van Eck & Waltman, Leiden University, Leiden, The Netherlands).

## 2. Materials and Methods

### 2.1. Data Collection

Studies were identified in the WoS databased comprising SCI-EXPANDED, SSCI, A&HCI, CPCI-S, CPCI-SSH, BKCI-S, BKCI-SSH, ESCI, and CCR-EXPANDE on 1 July 2021. The data retrieval strategy included the topics (perineal pain) AND the title (NP), and the publications between 1981 and 2021 were identified. The retrieval strategy was as follows: Perineal pain (Topic) and Articles or Review Articles or Proceedings Papers or Editorial Materials or Letters (Document Types) and 2021 or 2020 or 2019 or 2018 or 2017 or 2016 or 2015 or 2014 or 2013 or 2012 or 2011 or 2010 or 2009 or 2008 or 2007 or 2006 or 2005 or 2004 or 2003 or 2002 or 2001 or 2000 or 1999 or 1998 or 1997 or 1996 or 1995 or 1994 or 1993 or 1992 or 1991 or 1990 or 1989 or 1988 or 1986 or 1984 or 1983 or 1982 or 1981 (Publication Years).

### 2.2. Analysis Tool

Using the intrinsic function of WoS, we analyzed trends in research publications and the quality of publications from 1981 to 2021. We analyzed multiple parameters, including the country/region, institution distribution, and the top 10 cited references.

To mine and cluster data from retrieved articles, we also utilized VOSviewer and Microsoft Excel 2013 (Microsoft Corporation, Redmond, WA, USA. The software uses circles of different colors and sizes to describe authors, keywords, and countries based on how often they appear in headlines and summaries.

## 3. Results

### 3.1. Overview of Identified Papers

**Figure 1:**
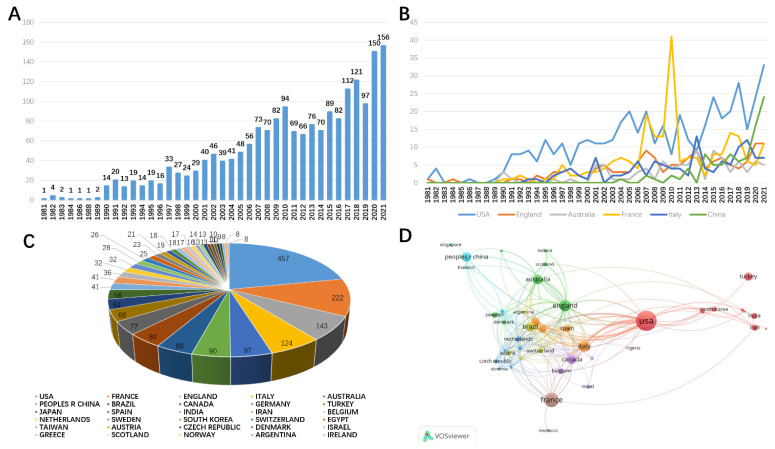
Overview of perineal pain-related articles. (**A**) The number of perineal pain publications across the world from 1981 to 2021. (**B**) The time curve of perineal pain-related articles from the top six countries. (**C**) The number of articles from each country/region. (**D**) A network map showing the collaborative relationships between various countries in the field of perineal pain research.

In the WOS database, the first article on perineal pain was published in 1981. From 1981 to 2021, a total of 1917 articles were included in the WOS database. The number of related papers published each year showed a significant increase year by year, which indicates that research on perineal pain has increased in the past 40 years. We also found that researchers from nearly 81 countries have published on perineal pain. Overall, authors from the United States published the most papers (457, 23.839%), followed by France (222, 11.581%), England (143, 7.46%), Italy (124, 6.468%), and Australia (97, 5.06%). The number of articles from China has grown rapidly in recent years, ranking second in the world for the past two years (Figure 1A–C).

We also analyzed the collaboration between authors from different countries. We found that authors from that 47 countries had published more than five papers, and we also identified seven closely related groups (seven clusters) in the field.

Among them, authors from the United States, Turkey, India, and South Korea appear to be closely connected. Authors from England, Australia, New Zealand, and Scotland also work closely together. Authors from China appear to work closely with authors from Singapore and Thailand. In summary, cross-country communication is common in perineal pain research. The US remains at the center of this research field, and China is playing an increasingly important role in recent years (Figure 1D).

### 3.2. Institutions and Author Collaborations

**Figure 2:**
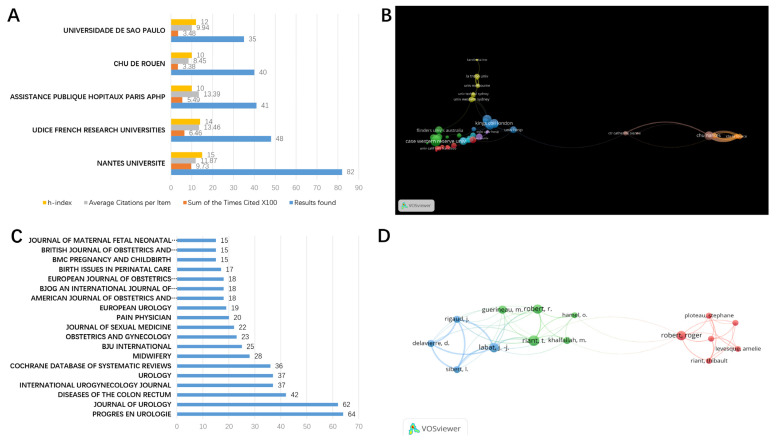
Institutions and author collaborations. (**A**) The total citations, average citations per paper, and H-indexes for perineal pain articles from institutions across the world. (**B**) The collaboration network of institutes with a notable interest in perineal pain research. (**C**) The number of articles from each journal. (**D**) The collaboration network of authors publishing research on perineal pain.

Our findings indicate that French academic institutions dominate the field of perineal pain. Authors from Nantes Universite published the most articles and had the highest H-index and total citations, reflecting a prominent position in the field. The article titled “Anatomic basis of chronic perineal pain: role of the pudendal nerve” by Robert et al. published in 1998 has been cited 167 times. Using VOSViewer software, we also identified multiple collaborations between French academic institutions, such as Nantes Universite and Udice French Research Universities. Authors from British, American, and Australian institutions also communicate frequently in the field of perineal pain (Figure 2A,B).

Progres en Urologie, a French journal, has published the most papers on perineal pain (64, 3.339%), indicating the importance of France in the research field. In line with the institutions that publish the most papers, Jean-Jacques Labat from Nantes Universite is the most published author in the field (Figure 2C,D). The Journal of Urology ranked second with 62 papers, which is a top journal in the field of urology. As the Official Journal of the American Urological Association (AUA), the Journal of Urology is the most widely read and highly cited journal in the field. This journal brings solid coverage of the clinically relevant content needed to stay at the forefront of the dynamic field of urology. Considering the complexity and the difficulty of perineal pain, many researchers are proud to publish related papers in this top journal. Of course, the Journal of Urology would like to pay more attention to this field.

### 3.3. Research Direction and Types of Language

**Figure 3:**
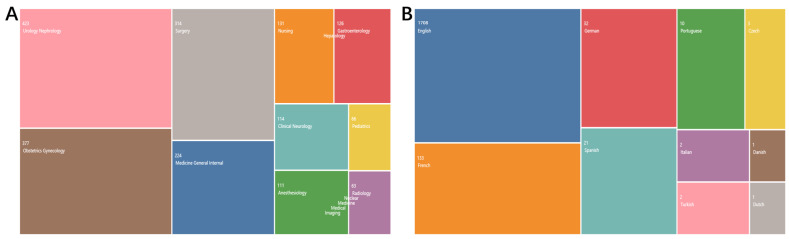
Research direction and language of published studies. (**A**) Research direction. (**B**) Language.

Most of the papers on perineal pain are clinical studies for which the research focus was mainly urology/nephrology and obstetrics/gynecology. Articles published in the French language were also frequently identified, which is due to the abundance of papers published in Progres en Urologie (Figure 3A,B). These results also reflect the importance of French research institutions and scholars in the field of perineal pain research.

### 3.4. The Top 10 Most Cited Articles on Perineal Pain

Table 1 shows the most cited papers identified in our review The most cited articles were reviews published in the Cochrane Database of Systematic Reviews. The paper titled “Local oestrogen for vaginal atrophy in postmenopausal women”, written by Suckling et al., is the most cited research paper in the past 40 years. Vaginal atrophy is a frequent complaint of postmenopausal women; symptoms include vaginal dryness, itching, discomfort, and perineal pain. We found that the most highly cited articles report on specific causes of perineal pain, and there is a lack of studies on unexplained perineal pain. The paper titled “Prevalence of prostatitis-like symptoms in a population based study using the National Institutes of Health chronic prostatitis symptom index” had been cited 300 times, ranking 2nd in the past 40 years. For men, chronic prostatitis is often the lead cause of perineal pain.

### 3.5. Keywords Co-Occurrence Analysis

**Figure 4:**
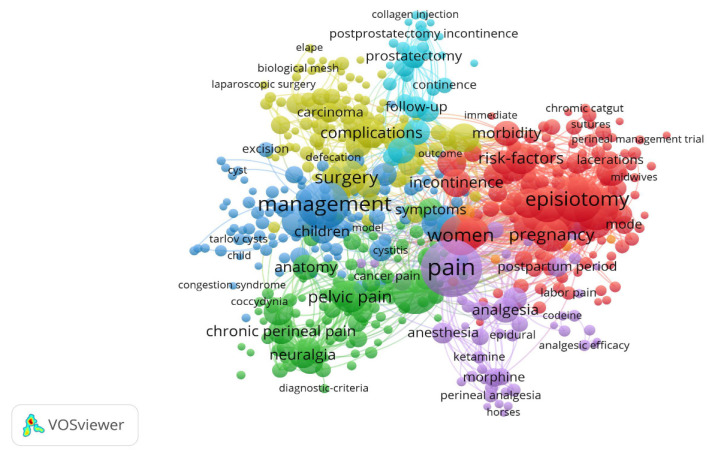
Keywords co-occurrence analysis. The network map of keywords related to perineal pain.

Using the VOSviewer software, we identified keywords that appear most frequently in studies of perineal pain. We analyzed papers to identify keywords that were listed in at least five studies. Of 6010 words identified overall, 531 met this threshold. Figure 4 shows that the 531 words could be classified into seven clusters: episiotomy, pain, management, prostatectomy, surgery, pelvic pain, and complication. From these results, we conclude that research on perineal pain is focused on clinical studies and that pain after surgery is a key focus. There is a marked lack of research on unexplained perineal pain.

## 4. Discussion

In this bibliometric analysis on perineal pain, we summarized and analyzed research trends and hot topics in the field.

Over the last 40 years, the number of papers on perineal pain increased rapidly worldwide, although the total number of articles is fewer than that seen for other medical disciplines. Authors from the United States and France have published extensively in the field. In particular, numerous studies on perineal pain have been published in the French-language journal “Progres en Urologie”.

In 2021, China ranked second in the world in terms of the number of published papers, after the United States. There is reason to believe that as the Chinese government invests more extensively in science and technology, and cooperation and collaboration between Chinese institutions and academic institutions in other countries will become more frequent.

From the perspective of author contributions, Jean-Jacques Labat and Robert Roger from Nantes, France, were the most published authors with H-indexes of 17 and 14, respectively. These authors jointly published “Diagnostic criteria for pudendal neuralgia by pudendal nerve entrapment (Nantes criteria) [1]”, which has been cited 217 times and provides a reference for the diagnosis of perineal pain. In addition, they also pioneered the use of techniques to treat perineal pain. For example, they published a prospective study of 27 consecutive cases on the effects of spinal cord stimulation of the conus medullaris for refractory pudendal neuralgia [9]. This study is the first to analyze the application of spinal cord stimulation for perineal pain. Jean-Jacques Labat and Robert Roger also participated in the development of an expert consensus on perineal pain. This expert consensus included recommendations for the diagnosis and treatment of pudendal nerve entrapment (PNE) syndrome, including drug treatments [10,11], physiotherapy [12], psychotherapy [13], injections, surgery [14], pulsed radiofrequency [15,16], and neuromodulation [2], and is the most comprehensive expert consensus on perineal pain to date. Besides these, some researchers also reported that motor cortex stimulation constitutes a new treatment for refractory pelvic and perineal pain and should be considered after failure of conventional neuromodulation techniques, especially spinal cord stimulation and motor cortex stimulation [17]. The mechanism of action of motor cortex stimulation involves various structures and neuronal pathways involved in pain regulation. Motor cortex stimulation initially activates axons, which run horizontally in the anterior central gyrus, parallel to the cortical surface. However, the biological effects of the activation of these neural circuits may be located at a distance from the site of the stimulus. Of course, we need more prospective randomized studies using transcranial magnetic stimulation and implanted motor cortex stimulation in larger populations to confirm the favorable results.

We also identified keywords that were most frequently included in relevant papers. We found that “episiotomy” and “prostatectomy” were mentioned in many articles on perineal pain, reflecting that perineal pain is a common postoperative complication associated with these procedures. Margarita Manresa et al. reported that perineal muscle trauma can result in perineal pain and dyspareunia lasting up to 10 days and 6 months postpartum, respectively [18]. In addition, a questionnaire showed that one third of patients who undergo radical retropubic prostatectomy develop chronic perineal pain, manifested as a burning sensation and numbness [19]. Of course, this chronic perineal pain induced by radical retropubic prostatectomy can be clinically confirmed via perineal electromyography eventually. For patients with perineal pain who have no history of surgery, diagnosis and treatment are particularly important.

We also found that “neuralgia” and “diagnosis-criteria” were frequently included as keywords. In the latest consensus on perineal pain, experts in the field have come up with a new diagnostic criteria, termed the Nantes criteria [1]. Per this system, a person with four clinical criteria and one invasive criterion (positive test for block after local anesthetic injection of the sciatic spine) can be diagnosed with PNE. Importantly, the long-term analgesic effect of corticosteroid injection cannot be concluded from existing data [20]. As such, experts do not recommend it for therapeutic purposes. Current treatment options do not cure perineal pain. Existing studies have shown that pudendal nerve release surgery is considered a long-term effective treatment for appropriate surgical candidates who meet the five Nantes criteria. The management and treatment of perineal pain is multi-channel, but the ultimate goal is to maximize functional recovery and significantly reduce the severity and intensity of the pain. Treatment includes drug therapy, nerve block and minimally invasive therapy, surgical nerve decompression, physical therapy, and psychotherapy, etc., but currently there is a lack of treatment standards and radical treatment. A combination of drugs, such as non-steroidal anti-inflammatory drugs, tricyclic antidepressants, anticonvulsants, and narcotic analgesics, is usually used. If the medication does not work, local nerve blocks or neuromodulation can be further administered to reduce perineal pain. Given the few studies concerning the use of pulsed radiofrequency and spinal cord stimulation in the context of PNE and potential morbidity, experts did not recommend these strategies for first-line treatment. However, given their proven efficacy in other indications for chronic pain, these techniques may be utilized for patients for whom surgery is either ineffective or not possible.

Our study has some limitations. Firstly, bibliometrics analysis, as a purely quantitative method, reflects more on the acceptance of an article in this field than on quality and the impact on future research. Therefore, more diverse assessment methods, such as a review and methodological analysis, may be combined with bibliometric parameters to provide more comprehensive insight into the research dynamics. Secondly, we only searched the WOS database; therefore, it is possible that not all relevant papers were identified. Thirdly, considering that groin pain is not entirely perineal pain, we can only cover it briefly in the discussion section. Our bibliometric analysis can not fully include groin pain, so research on perineal pain is also biased.

## 5. Conclusions

In conclusion, the number of related papers published showed a significant increase year by year, which indicates that perineal pain has become more widely studied in the past 40 years. Studies the from US and France were most frequently identified. In addition, the number of studies from China has increased rapidly in recent years, ranking second in the world in the past two years. The French journal Progres en Urologie has published the most papers on perineal pain. Meanwhile, the expert consensus led by French institutions and authors has provided invaluable insight into the diagnosis and treatment of perineal pain.

This analysis comprehensively reflects the research status of perineal pain and key developments and trends in the field, which may help to guide researchers when designing research questions and studies in the future.

## Figures and Tables

**Table 1:** The top 10 most cited articles regarding perineal pain in the world.

Title	Authors	Publication Date	Source Title	Total Citations
Local Oestrogen for Vaginal Atrophy in Postmenopausal Women	Suckling, J.; Lethaby, A.; Kennedy, R.	2006	Cochrane Database of Systematic Reviews	324
Prevalence of Prostatitis-Like Symptoms in a Population Based Study Using the National Institutes of Health Chronic Prostatitis Symptom Index	Nickel, J.C.; Downey, J.; Hunter, D.; Clark, J.	March 2001	Journal of Urology	300
Maternal Health After Childbirth: Results of an Australian Population Based Survey	Brown, S.; Lumley, J.	February 1998	British Journal of Obstetrics and Gynaecology	278
Women’s Sexual Health After Childbirth	Barrett, G.; Pendry, E.; Peacock, J.; Victor, C.; Thakar, R.; Manyonda, I.	February 2000	British Journal of Obstetrics And Gynaecology	271
Episiotomy for Vaginal Birth	Carroli, G.; Mignini, L.	2009	Cochrane Database of Systematic Reviews	270
Relationship of Episiotomy to Perineal Trauma and Morbidity, Sexual Dysfunction, and Pelvic Floor Relaxation	Klein, M.C.; Gauthier, R.J.; Robbins, J.M.; Kaczorowski, J.; Jorgensen, S.H.; Franco, E.D.; Johnson, B.; Waghorn, K.; Gelfand, M.M.; Guralnick, M.S.; Luskey, G.W.; Joshi, A.K.	September 1994	American Journal of Obstetrics and Gynecology	256
Prevalence and Persistence of Health Problems after Childbirth: Associations with Parity and Method of Birth	Thompson, J.F.; Roberts, C.L.; Currie, M.; Ellwood, D.A.	June 2002	Birth-Issues in Perinatal Care	253
The Use of Ultrasound Imaging of the Abdominal Drawing-in Maneuver in Subjects with low Back Pain	Teyhen, D.S.; Miltenberger, C.E.; Deiters, H.M.; Del Toro, Y.M.; Pulliam, J.N.; Childs, J.D.; Boyles, R.E.; Flynn, T.W.	June 2005	Journal of Orthopaedic & Sports Physical Therapy	252
Posterior Colporrhaphy: Its Effects on Bowel and Sexual Function	Kahn, M.A.; Stanton, S.L.	January 1997	British Journal of Obstetrics and Gynaecology	247
Outcomes of Routine Episiotomy—A Systematic Review	Hartmann, K.; Viswanathan, M.; Palmieri, R.; Gartlehner, G.; Thorp, J.; Lohr, K.N.	4 May 2005	JAMA-Journal of the American Medical Association	242

## Data Availability

The datasets analyzed during the current study are available from the corresponding author on reasonable request.

## References

[1] Labat J.J., Riant T., Robert R., Amarenco G., Lefaucheur J.P., Rigaud J. (2008). Diagnostic criteria for pudendal neuralgia by pudendal nerve entrapment (Nantes criteria). Neurourol. Urodyn..

[2] Levesque A., Bautrant E., Quistrebert V., Valancogne G., Riant T., Beer Gabel M., Robert R. (2022). Recommendations on the management of pudendal nerve entrapment syndrome: A formalised expert consensus. Eur. J. Pain.

[3] Zhang Y., Yin P., Liu Y., Hu Y., Hu Z., Miao Y. (2022). Global trends and hotspots in research on organoids between 2011 and 2020: A bibliometric analysis. Ann. Palliat. Med..

[4] Liu Y., Ding L., Xianyu Y., Nie S., Yang J. (2022). Research on depression in Parkinson disease: A bibliometric and visual analysis of studies published during 2012–2021. Medicine.

[5] Zhao T., Hu H., Chen X., Liu Y., Wang H., Li X. (2022). Mapping trends and hotspots regarding oral carcinoma and macrophages: A bibliometric analysis of global research. Am. J. Transl. Res..

[6] Hacad C.R., Lucon M., Milhomem S., Bruschini H., Tanaka C. (2022). Association of physical therapy techniques can improve pain and urinary symptoms outcomes in women with bladder pain syndrome. A randomized controlled trial. Int. Braz. J. Urol..

[7] Hou J., Su H., Kuang X., Qin W., Liu K., Pan K., Hua Q. (2022). Knowledge Domains and Emerging Trends of Osteoblasts-Osteoclasts in Bone Disease From 2002 to 2021: A Bibliometrics Analysis and Visualization Study. Front. Endocrinol..

[8] Lei K., Wang X., Liu Y., Sun T., Xie W. (2022). Global research hotspots and trends of the Notch signaling pathway in the field of cancer: A bibliometric study. Am. J. Transl. Res..

[9] Buffenoir K., Rioult B., Hamel O., Labat J.J., Riant T., Robert R. (2015). Spinal cord stimulation of the conus medullaris for refractory pudendal neuralgia: A prospective study of 27 consecutive cases. Neurourol. Urodyn..

[10] Levesque A., Riant T., Labat J.J., Ploteau S. (2017). Use of High-Concentration Capsaicin Patch for the Treatment of Pelvic Pain: Observational Study of 60 Inpatients. Pain Physician.

[11] Levesque A., Ploteau S., Michel F., Siproudhis L., Bautrant E., Eggermont J., Labat J.J. (2021). Botulinum toxin infiltrations versus local anaesthetic infiltrations in pelvic floor myofascial pain: Multicentre, randomized, double-blind study. Ann. Phys. Rehabil. Med..

[12] Levesque A., Riant T., Ploteau S., Rigaud J., Labat J.J., Convergences P.P. (2018). Network. Clinical Criteria of Central Sensitization in Chronic Pelvic and Perineal Pain (Convergences PP Criteria): Elaboration of a Clinical Evaluation Tool Based on Formal Expert Consensus. Pain Med..

[13] Origo D., Tarantino A.G. (2019). Osteopathic manipulative treatment in pudendal neuralgia: A case report. J. Bodyw. Mov. Ther..

[14] Ploteau S., Perrouin-Verbe M.A., Labat J.J., Riant T., Levesque A., Robert R. (2017). Anatomical Variants of the Pudendal Nerve Observed during a Transgluteal Surgical Approach in a Population of Patients with Pudendal Neuralgia. Pain Physician.

[15] Masala S., Calabria E., Cuzzolino A., Raguso M., Morini M., Simonetti G. (2014). CT-guided percutaneous pulse-dose radiofrequency for pudendal neuralgia. Cardiovasc. Intervent. Radiol..

[16] Le Clerc Q.C., Riant T., Levesque A., Labat J.J., Ploteau S., Roger R., Rigaud J. (2017). Repeated Ganglion Impar Block in a Cohort of 83 Patients with Chronic Pelvic and Perineal Pain. Pain Physician.

[17] Louppe J.M., Nguyen J.P., Robert R., Buffenoir K., de Chauvigny E., Riant T., Nizard J. (2013). Motor cortex stimulation in refractory pelvic and perineal pain: Report of two successful cases. Neurourol. Urodyn..

[18] Manresa M., Pereda A., Goberna-Tricas J., Webb S.S., Terre-Rull C., Bataller E. (2020). Postpartum perineal pain and dyspareunia related to each superficial perineal muscle injury: A cohort study. Int. Urogynecol. J..

[19] Turmel N., Ismael S.S., Chesnel C., Charlanes A., Hentzen C., Le Breton F., Amarenco G. (2019). Use of a specific questionnaire and perineal electromyography to assess neuropathic pain after radical retropubic prostatectomy. Asian J. Urol..

[20] Labat J.J., Riant T., Lassaux A., Rioult B., Rabischong B., Khalfallah M., Ploteau S. (2017). Adding corticosteroids to the pudendal nerve block for pudendal neuralgia: A randomised, double-blind, controlled trial. BJOG.

